# Identification of cell cycle-related regulatory motifs using a kernel canonical correlation analysis

**DOI:** 10.1186/1471-2164-10-S3-S29

**Published:** 2009-12-03

**Authors:** Je-Keun Rhee, Je-Gun Joung, Jeong-Ho Chang, Zhangjun Fei, Byoung-Tak Zhang

**Affiliations:** 1 Graduate Program in Bioinformatics, Seoul National University, Seoul 151-744, Korea; 2 Center for Biointelligence Technology (CBIT), Seoul National University, Seoul 151-744, Korea; 3 Boyce Thompson Institute for Plant Research, Cornell University, Ithaca, NY 14853, USA; 4 Konan Technology Inc., Seoul 135-080, Korea; 5 USDA Robert W. Holley Center for Agriculture and Health, Ithaca, NY 14853, USA; 6 School of Computer Science and Engineering, Seoul National University, Seoul 151-744, Korea

## Abstract

**Background:**

Gene regulation is a key mechanism in higher eukaryotic cellular processes. One of the major challenges in gene regulation studies is to identify regulators affecting the expression of their target genes in specific biological processes. Despite their importance, regulators involved in diverse biological processes still remain largely unrevealed. In the present study, we propose a kernel-based approach to efficiently identify core regulatory elements involved in specific biological processes using gene expression profiles.

**Results:**

We developed a framework that can detect correlations between gene expression profiles and the upstream sequences on the basis of the kernel canonical correlation analysis (kernel CCA). Using a yeast cell cycle dataset, we demonstrated that upstream sequence patterns were closely related to gene expression profiles based on the canonical correlation scores obtained by measuring the correlation between them. Our results showed that the cell cycle-specific regulatory motifs could be found successfully based on the motif weights derived through kernel CCA. Furthermore, we identified co-regulatory motif pairs using the same framework.

**Conclusion:**

Given expression profiles, our method was able to identify regulatory motifs involved in specific biological processes. The method could be applied to the elucidation of the unknown regulatory mechanisms associated with complex gene regulatory processes.

## Background

One of the major challenges in current biology is to elucidate the mechanism governing the gene expression. Gene expression programs depend mainly on transcription factors which bind to upstream sequences by recognizing short DNA motifs called transcription factor binding sites (TFBSs) to regulate their target gene expression [1]. Although many regulatory motifs have been identified, large amount of functional elements still remain unknown [2].

Many genome-wide approaches have been developed in attempt to discover regulatory motifs from upstream sequences. The early computational approach for identifying regulatory motifs is based on statistical analyses using only upstream sequences of genes. Statistical methods such as maximum-likelihood estimation or Gibbs sampling, are effective for searching directly significant sequence motifs from multiple upstream sequences [3,4]. Several computational approaches based on machine learning methods have also been implemented. A SOM (self-organizing map)-based clustering method can find regulatory sequence motifs by grouping relevant sequence patterns [5] and a graph-theoretic approach has tried to identify regulatory motifs by searching the maximum density subgraph [6].

More advanced approaches have been developed that can identify regulatory motifs by linking gene expression profiles and motif patterns. The main advantage of these approaches is that they can identify motifs correlated to specific biological processes. Most early trials used a unidirectional search, such as approaches that search for shared patterns with upstream sequences in a set of co-expressed genes that were found by clustering algorithms [7,8] or those that determine whether genes with common regulatory elements are co-expressed [9,10]. In addition, it is also possible to link motifs to gene expression patterns using linear regression models or regression trees [11,12]. Recently, several techniques for a bidirectional search to detect the relationship between the regulatory motifs and the gene expression profiles have been emerged [13,14]. They search regulatory motifs more efficiently than unidirectional approaches since they search similar expression patterns and regulatory motifs correlated to them simultaneously.

**Figure 1:**
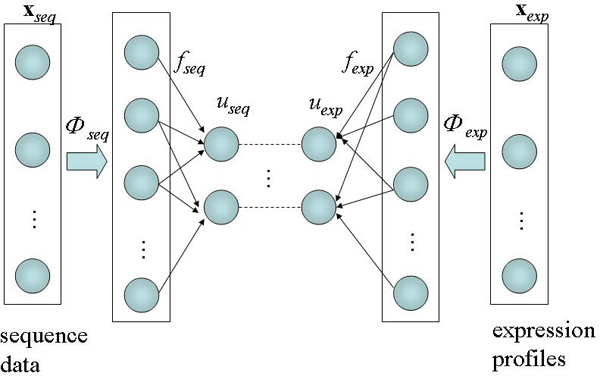
**Basic scheme of the kernel CCA**. The sequence and expression data are transformed to Hilbert space by *φ *function. By taking inner products, *u*_*exp *_and *u*_*seq *_were derived, which maximize the correlation between the upstream sequences and the expression profiles.

In this study, we propose a novel bidirectional approach using a kernel-based method, kernel CCA (kernel canonical correlation analysis), to analyze the relationship between regulatory sequences and gene expression profiles [15-17]. The expression and sequence features are mapped from the original input space to a higher dimension space using a kernel trick, and the relationship between the two projected objects is interpreted to identify highly correlated motifs (Figure 1). Our method has advantages that it can detect core motifs relevant to a specific cellular process without the additional efforts of clustering and intensive motif sampling process in upstream sequences.

We applied the kernel CCA to a paired set of upstream sequence motifs of genes and their expression profiles in yeast (*Saccharomyces cerevisiae*) cell cycle, and explored significant relationships between motifs and expression profiles. We also searched for regulatory motifs correlated with specific expression patterns. Our method retrieved regulatory motifs that play an important role in cell cycle regulation including several well-known cell cycle regulatory motifs: MCB, SCB and SFF'. Furthermore, we identified motif pairs associated with the gene expression to construct a map of combinatorial regulation of regulators.

## Results and discussion

We applied a computational method, kernel CCA, to the identification of novel transcriptional regulatory elements. The main purpose of our experiments was to find regulatory motifs that were associated with gene regulation in specific biological processes. Using the kernel CCA, we first found highly correlated features between expression profiles and the sequence motifs. The key motifs in gene regulation were then identified from the weight scheme by the kernel CCA (see Methods section). Furthermore we demonstrate that it is possible for our method to be applied for identification of motif pairs using raw upstream sequences.

### Identification of the relationship between gene expression and known motifs

We first explored the relationship between gene expression profiles and known motifs using a yeast gene expression dataset related to the cell cycle [18] and a set of known motifs (Table 1) extracted by AlignACE [9]. A total of 551 ORFs (open reading frames) in the expression dataset contained at least one known motif. In the parameter setting, the degree of polynomial kernel was set to 3, the parameter *σ *in Gaussian RBF kernel was 0.5, and the regularization parameter was 0.1. These parameters were chosen based on the parameter setting that produced a high correlation from multiple runs.

**Table 1:** Known regulatory motifs in yeast (*Saccharomyces cerevisiae*)

Motif			
RAP1	RPN4	GCN4	MCB
HAP234	MIG1	AFT1	STRE'
CCA	CSRE	PHO4	STE12
HSE	ABF1	ATRepeat	GAL
Leu3	LYS14	MET31-32	OAF1
PAC	PDR	PHO	REB1
STRE	ECB	ndt80 (MSE)	Yap1
SCB	Gcr1	zap1	MCM1'
MCM1	SFF	SFF'	BAS1
Ume6 (URS1)	SWI5	ALPHA1'	ALPHA1
ALPHA2'	ALPHA2		

**Figure 2:**
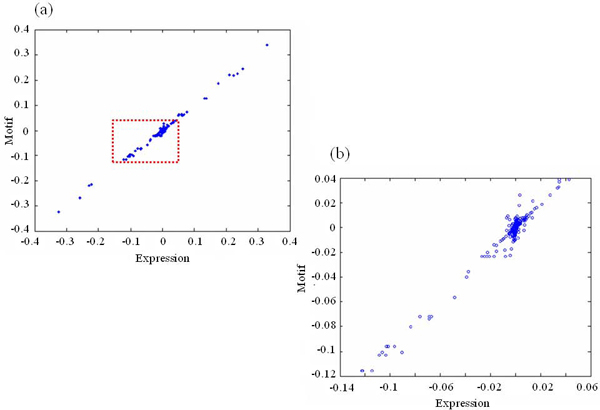
**Relationship between gene expression profiles and regulatory sequence motifs**. (a) The plot shows the correlation between gene expression profiles and the regulatory sequence motifs. Each dot represents one gene in the dataset, and x-axis means the value of *u*_*exp*_, y-axis is *u*_*seq*_. (b) The plot is a close-up view of the boxed area in (a).

The results from the kernel CCA were visualized using the CC1 (first canonical correlation) score (Figure 2). In Figure 2, each point corresponds to a gene, and a cloud of the diagonal points illustrated the correlation between the expression and the motifs. The shape of diagonal points and the high correlation coefficient (0.996) indicated that the kernel CCA was able to find the close relationship between the expression profiles and the sequence motifs. We then performed the linear canonical correlation analysis using the same datasets. The correlation coefficient (0.612) obtained from the linear CCA was much lower. As shown in Additional file 1, the linear CCA could not identify the significant correlation between expression profiles and motifs. This further supports that kernel CCA improve significantly in finding the correlation between the two datasets.

The motifs were searched by the weight function of Equation 6 (see Methods section) with the model obtained by the kernel CCA and the top ranked motifs are shown in Table 2. SWI5 motif, a binding site of SWI5 protein, has the highest weight value. SWI5 has been known to act in G1 phase and in the M/G1 boundary in the cell cycle [19,20]. SFF' motif is a binding site of FKH1 transcription factor that affects the expression of genes controlling the cell cycle during the G2-S phase change [21]. The MCB motif is one of the well-known motifs in the yeast cell cycle as a binding site in the MBF protein complex. MBF protein is composed of MBP1 and SWI6, and MBP1 is a DNA binding component while SWI6 has regulatory roles. It is well known that the MBF protein complex regulates the transcription of many genes in the late G1 phase [19,22]. ALPHA2 protein also plays a role in the cell cycle. It operates synergistically with MCM1 protein to repress the expression of its target genes [23,24]. MCM1 protein is a key regulator involved in the transcription of several M/G1 genes during the cell cycle [10,22,25]. A high weight value of ALPHA2 is supported by the evidence that ALPHA2 protein binds to the MCM1 protein and influences the regulation of other cell cycle-related genes [26,27]. Using the set of known motifs, our results are consistent with previous reports, validating the analysis method employed.

**Table 2:** The list of top ranked motifs based on the weight scheme by the kernel CCA

Motif	Weight	Function	Reference
SWI5	0.89026	Transcription activation in G1 phase	[19,20]
SFF'	0.45399	FKH1 binding site that regulate the cell cycle	[21]
MCB	0.29633	MBF binding site that activates in late G1 phase	[19,22]
LYS14	0.21796	Lysine biosysthesis pathway	
ALPHA2	0.16532	Encoding a homeobox-domain	[23,24]

**Figure 3:**
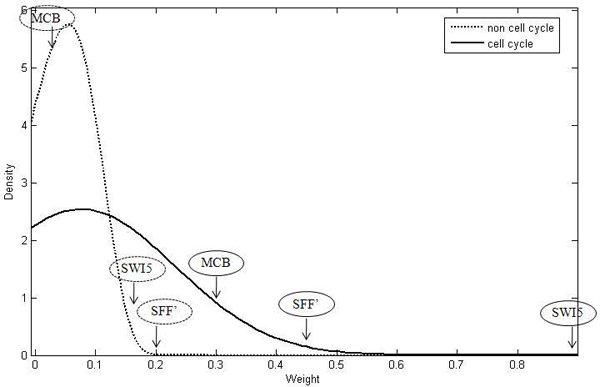
**Weight distributions for MCB, SFF' and SWI5 motifs derived from cell cycle and non cell cycle-related datasets**. The dotted line indicates the weight distribution from the non-cell cycle datasets and the solid line from cell cycle datasets.

To further validate the result of top-ranked motifs extracted by kernel CCA, we compared the weights obtained from cell cycle-related ORF set with those obtained from randomly selected set. We performed the same procedure using random ORFs that are not known to be related to the cell cycle. Figure 3 shows the highly weighted motifs obtained from our method in cell cycle-related gene set and non cell cycle set, and the relative positions of those motifs are presented in the weight distribution of all motifs. The weight values obtained from random set were significantly lower than those obtained from cell cycle-related ORF set. We could infer that the significantly correlated motifs were not extracted from these random datasets. In summary, our method could identify the regulatory motifs that have high weights indicating high correlation between the upstream sequences and the gene expression profiles.

### Identification of cell cycle-related motifs

**Figure 4:**
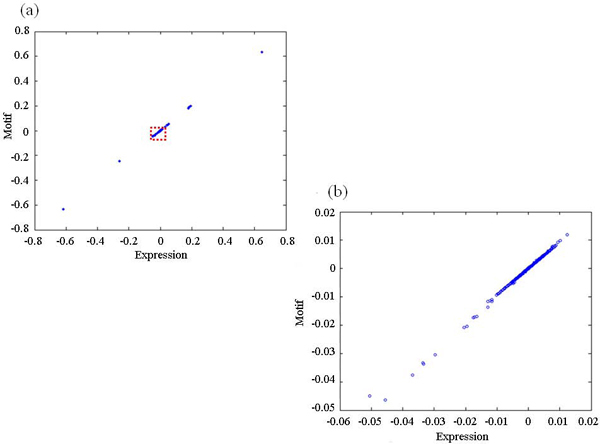
**Correlation between expression profiles and motifs derived by using the raw upstream sequence data**. The plot on (b) is an enlargement of the boxed area in (a).

We then applied the linear kernel to the motif sequence data containing a total of 1,024 features (window size *l *= 5) extracted from the raw upstream sequences of genes and Gaussian RBF kernels with parameter *σ *values of 0.3 to the expression data. The regularization parameter was set to 0.1. These parameters are also empirically chosen based on the fact that they produced a high correlation. Figure 4 shows the CC1 score which represents the correlation between the expression profiles and the sequence patterns. When the linear kernel was applied to the sequence dataset, the expression data is closely related to the motif data using the raw sequences of 5-mers.

The 5-mer motif patterns with high weights are listed in Table 3. The 5-mer with the highest weight is 5'-GCGTG-3', which is similar to the MCB motif (5'-ACGCGT-3'). As described previously, MCB is an important motif involved in the cell cycle. The second-ranked sequence (5'-CGTGT-3') matched to the first five bases of the ALPHA2 motif sequence. From the second component, we also found several significant sequences, including a consensus sequence (5'-CGCGT-3') that is identical to the MCB motif (5'-ACGCGT-3'). This further confirmed that the MCB motif affects gene expression in the cell cycle. Another interesting motif is 5'-CCACG-3', which is a sequence block with one base shift from the known SCB motif (5'-CACGAAA-3'). The SCB motif is a binding site of the SBF protein, which is a complex of SWI4 (a DNA-binding component) and SWI6 (a regulatory component) [22], and SBF is a major regulator in the G1/S transition. In each component, the list of 100 motif patterns with high weights is provided in Additional file 2.

**Table 3:** High-scored motifs in the first and the second components using 5-mer raw upstream sequences

Sequence	Motif Description	Weight	Component	Rank
GCGTG	MCB (ACGCGT)	0.079567	1	1
CGTGT	MATalpha2 (CRTGTWWWW)	0.075340	1	2
CATGT	MATalpha2 (CRTGTWWWW)	0.046299	1	12
CCACG	SCB (CACGAAA)	0.018992	2	4
CGCGT	MCB (ACGCGT)	0.017870	2	5
GTGTT	MATalpha2 (CRTGTWWWW)	0.016595	2	9

### Combinational effects of regulatory motifs

We searched the motif pairs that have synergistic or co-regulatory combination effects in the yeast cell cycle. The regulatory mechanisms of eukaryotes are highly complex since most genes are normally synergistically regulated by different transcription factors. Therefore, identifying the synergistic motif combinations can contribute to systematically understanding the regulatory circuit.

In the present study, using the kernel CCA we calculated the weight value for each motif pair of 42 known motifs. The heat map of weight values of all motif pairs is provided in Additional file 3. Table 4 presents the top ten motif pairs with the highest weight values and with occurrence of more than ten in all the investigated upstream sequences. It also shows ECRScores which represent gene expression coherence. All these scores are relatively high compared to the previously identified synergistic motif pairs (ECRScores > 0.075) [9]. As shown in Table 4, the pair with the highest weight value is MCB-MCM1. According to a previous study, MCB and MCM1 were characterized as a significantly cooperative motif pair in the regulation of the cell cycle [28]. Other highly ranked pairs, such as ECB-ALPHA2 and MCM1-ALPHA2, are already known that they are required for transcriptional regulation of early cell cycle genes. MCM1 activates transcription of ECB (early cell cycle box)-dependent genes during M/G1 phase [29], and the MCM1 protein can interact with the ALPHA2 factor regulating the expression of mating-type-specific genes [26,27]. These evidences support that two ALPHA2-related motif pairs act synergistically in the expressional regulation of the yeast cell cycle process. The REB1 motif, a binding site of REB1 protein, is frequently found among the pairs of motifs with the highest weights. The REB1 protein is an RNA polymerase I enhancer-binding protein and binds to genes transcribed by both RNA polymerase I and RNA polymerase II [30]. It is a general regulator rather than a condition specific one. Therefore, it is reasonable that this protein shows a high frequency in our results. REB1-SWI5, REB1-MCM1' and REB1-ALPHA1 motif pairs are already identified as acting synergistically in the yeast cell cycle regulation [31-33]. Most of our results are consistent with the previous reports. In addition, it's worth noting that several previously uncharacterized motif pairs were identified by our kernel CCA methods.

**Table 4:** The top 10 ranked motif pairs and their ECRScores

Weight	Motif Pair		ECRScore	# of ORFs	Reference
2.5368	MCB	MCM1	0.390	15	[28]
2.5018	MCB	ECB	0.439	12	
2.0177	PHO	MCM1'	0.088	17	
1.848	ECB	ALPHA2	0.088	14	
1.7535	MCM1	ALPHA2	0.074	17	[26,27]
1.7263	ATRepeat	MCM1	0.076	12	
1.6995	PHO	ECB	0.127	11	
1.6823	REB1	SWI5	0.099	14	[31]
1.6476	REB1	MCM1'	0.115	13	[32,33]
1.4256	REB1	ALPHA1	0.067	15	[33]

## Conclusion

We presented a novel method that can identify the candidate conditional specific regulatory motifs by employing kernel-based methods. The application of the kernel CCA enables us to detect correlations between heterogeneous datasets, consisting of upstream sequences and expression profiles. From a data-mining perspective, our work is regarded as a new approach for detecting important features from regulatory sequences and gene expression profiles. We demonstrated that major motifs in a specific biological process can be extracted by a CC score via modelling a close relationship between two datasets related to gene regulation.

As genome-wide datasets of various types become available, it's important to analyze these datasets in an integrated manner [34]. It is possible to come up with novel biological hypotheses by integrating diverse biological resources generated for specific research purposes. In these aspects, the kernel CCA is regarded as a useful method that can extract the biological factors with significant roles by integrating different types of biological data. Many studies for identifying motifs have been based on sequence conservation or sequence characteristics, regardless of the biological processes. Therefore our method can be regarded as complementary approach in the analysis of gene regulation.

Our method found important motifs related to the cell cycle by using raw upstream sequences as well as known motif sets. In the present study we used the raw sequences of window size, *l *= 5. If we enlarged the window size, the dimension for sequence features increased exponentially, whereas the frequency of motifs decreased. Although the window size used in our experiments was shorter than the length of several known transcription factor binding sequences, it was long enough to obtain worthwhile results.

In the future research, we will apply the proposed method to diverse gene expression datasets, especially cancer-related datasets. The cancer-related regulatory program can be elucidated by analyzing regulatory motifs from a set of enriched genes in the cancer transcriptome [35]. Using the kernel CCA, a correlation analysis between regulatory sequences and the cancer transcriptome may directly catch regulatory motifs related to the abnormal gene regulatory program.

## Methods

### Investigation of the relationship between regulatory sequence motifs and expression profiles

Kernel CCA (Canonical correlation analysis) is a version of the nonlinear CCA, where the kernel trick is utilized to find nonlinearly correlated features from two datasets [15-17]. CCA is a classical multivariate statistical method for finding linearly correlated features from a pair of datasets [36]. Suppose there is a pair of multivariates **x **and **y**, CCA finds a pair of linear transformations such that the correlation coefficient between extracted features is maximized. However, if there is a nonlinear relationship between the variates, CCA does not always extract useful features.

Kernel CCA offers a solution for overcoming the linearity by first projecting the data into a higher dimensional feature space. While CCA is limited to linear features, kernel CCA can capture nonlinear relationships. Kernel CCA has been used for several applications including text retrieval and biological data analysis [15,37].

Figure 1 illustrates the basic scheme of the kernel CCA for our integrated analysis of DNA sequence motif and gene expression data. Using kernel CCA, we tried to find maximally correlated features between the gene expression and the sequence motifs. Here, a gene set **X **is represented by two separate profiles in terms of its transcriptional behaviour and upstream sequences, **x**_*exp *_and **x**_*seq*_. These are composed of the expression profile, **x**_*exp *_= (*e*_1_, *e*_2_, ..., *e*_*N*_) and the sequence profile, **x**_*seq *_= (*m*_1_, *m*_2_, ..., *m*_*M*_) of each gene. Here e_*i *_(1 ≤ *i *≤ *N*) is the expression value of the gene in the *i*-th sample or experimental condition from the microarray dataset, and m_*j *_(1 ≤ *j *≤ *M*) denotes the occurrence frequency of the *j*-th sequence motif in the upstream region of the gene. For the detection of correlated features between the two datasets, **x**_*exp *_and **x**_*seq *_are first mapped to Hilbert space, *H*, by function *φ*. That is, each **x **is projected into two directions, *f*_*exp *_and *f*_*seq*_, in Hilbert space according to its representation:

where ⟨•,•⟩ denotes the dot product. Kernel CCA looks for maximally correlated features between **x**_*exp *_and **x**_*seq*_:

where *λ*_*exp *_and *λ*_*seq *_are regularization parameters, var(•) means a variance and cov(•,•) is a covariance between two variables. The kernel CCA can be given by solving a generalized eigenvalue problem:

where **I **denotes the identity matrix, **K**_*exp *_is the kernel matrix for expression profiles, and **K**_*seq *_is the kernel matrix for sequence motifs. When given *α*_*exp *_and *α*_*seq *_as the solution of the above generalized eigenvalue problem with the largest eigenvalue, canonical correlation scores (CC scores) for **x**_*seq *_and **x**_*exp *_are estimated by *u*_*seq *_= **K**_*seq*_*α*_*seq *_and *u*_*exp *_= **K**_*exp*_*α*_*exp*_, respectively. The CC scores are based on the low dimensional-mapping of genes in terms of two separated representations and can be used to show the salient correlation between the two. Once we obtain the *α *vector, the weights of the motif and expression profile, **W**_*seq *_and **W**_*exp*_, are obtained as following:

A high weight value of the specific sequence motif means that the motif is strongly correlated with the expression patterns of genes whose upstream region includes the motif and whose CC scores are high. If a weight of a specific motif has a high absolute value, the motif is more likely to play a regulatory role in the specific biological process. The kernel CCA was implemented using Matlab.

### Preparation of the gene expression datasets

Expression profiles of all ORFs (open reading frames) during the yeast cell cycle that consists of 18 time points in the alpha factor synchronization case [18] were used as the expression dataset. To map from the expression profiles to high dimensional space, we converted them to the kernel matrix. We applied a gaussian RBF kernel to the expression profile matrix by:

where *σ *is a parameter and function *d*(•,•) is a Euclidean distance. The **x **and **x**' mean the two different instances.

### Preparation of the gene sequence datasets

The sequence data was used in two ways. In the first case, we used the sequences of a total of 42 known motifs (Table 1) extracted by Pilpel [9]. We then scanned the upstream regions of ORFs for the presence of these motifs using the AlignACE program [3]. The sequence profile was represented by the occurrence of these motifs in the promoters of each gene in the genome.

In the second case, we analyzed the relationship between the expression profiles and the raw upstream sequences. We extracted ~1 kb upstream sequences of each gene. From these sequences, we calculated the frequency of all possible *l*-mers in each gene. For *l *= 5, each gene had 1,024 (= 4^5^) different base combinations. The sequence profile was encoded in the frequency of *l*-mers.

We applied the kernel as  to the sequence data. When *d *= 1, it is the linear kernel, and when *d *> 1, it is the polynomial kernel.

### Measurement of the effect of motif pairs

To measure the effect of the motif pairs, we defined the ECRScore (Expression Coherence coRrelation Score) calculated by a Pearson correlation coefficient of expression profiles for all possible pairs of genes whose upstream regions had the two motifs, *m*_*i *_and *m*_*j*_:

where *N*(*m*_*i *_∩ *m*_*j*_) is the number of all pairs of genes whose upstream regions have the two motifs, and *N*_*τ*_(*m*_*i *_∩ *m*_*j*_) is the number of gene pairs whose correlation coefficient is larger than the threshold *τ*. The threshold was chosen based on the fifth percentile of the distribution for correlation coefficients of randomly sampled gene pairs.

## Competing interests

The authors declare that they have no competing interests.

## Authors' contributions

JKR implemented programs, built the experimental datasets, carried out the analysis and wrote the manuscript. JGJ developed the idea, led the analysis of the experimental results and wrote the manuscript. JHC was involved in the overall procedure of implementation. ZF contributed to the biological interpretation and wrote the manuscript. BTZ provided intellectual guidance and mentorship. All authors read and approved the final manuscript.

## Note

Other papers from the meeting have been published as part of *BMC Bioinformatics* Volume 10 Supplement 15, 2009: Eighth International Conference on Bioinformatics (InCoB2009): Bioinformatics, available online at http://www.biomedcentral.com/1471-2105/10?issue=S15.

## Supplementary Material

Additional file 1 Relationship between gene expression profiles and regulatory motifs from the linear CCA. Click here for file

Additional file 2 The top 100 ranked motifs in the first and the second components using possible 5-mer raw upstream sequences. Click here for file

Additional file 3 Heat map of weight values of motif pairs related to cell cycle regulation. Click here for file
