# Supplementary material for: Identification of cell cycle-related regulatory motifs using a kernel canonical correlation analysis
Source: BMC Genomics. 2009 Dec 3;10(Suppl 3):S29. doi: 10.1186/1471-2164-10-S3-S29 (PMC2788382; doi:10.1186/1471-2164-10-S3-S29)

### Additional file 1 –Relationship between gene expression profiles and regulatory motifs from the linear CCA


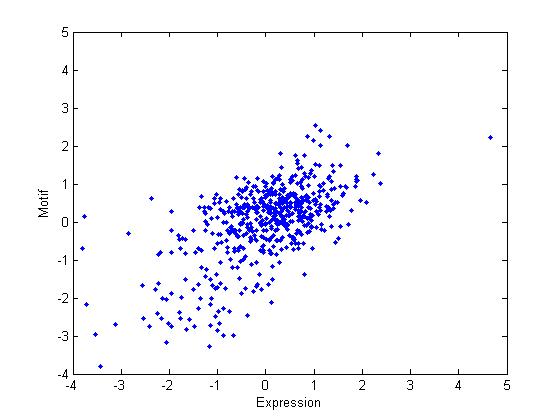

Supplement: Additional file 1 — Relationship between gene expression profiles and regulatory motifs from the linear CCA. [file 1471-2164-10-S3-S29-S1.doc]
